# JCMM Annual Review on Advances in Biotechnology for the Treatment of Haematological Malignancies: A Review of the Latest In‐Patient Developments 2024–2025

**DOI:** 10.1111/jcmm.70700

**Published:** 2025-07-06

**Authors:** Akbar M. Shahid, William Vainchenker, Stefan N. Constantinescu

**Affiliations:** ^1^ Ludwig Institute for Cancer Research University of Oxford Oxford UK; ^2^ INSERM UMR1287 Villejuif France; ^3^ Université Paris‐Saclay Villejuif France; ^4^ Gustave Roussy Villejuif France; ^5^ Ludwig Institute for Cancer Research Brussels Belgium; ^6^ Université Catholique de Louvain and de Duve Institute Brussels Belgium; ^7^ Walloon Excellence in Life Sciences and Biotechnology Brussels Belgium

**Keywords:** antibody‐drug conjugate, Bcl‐2 inhibitors, biotechnology, bispecific antibodies, cancer vaccines, epigenetics, gene therapy, haematological malignancies, monoclonal antibodies, protein degraders

## Abstract

Advances in biotechnology are set to revolutionise the treatment of haematological malignancies. Current treatments have limited efficacy due to their high toxicity and poor long‐term outcomes, particularly in acute myeloid leukaemia (AML). In 2024–2025, emerging targeted therapies promise improved results with reduced adverse effects. This review is the first of an annual series exploring the latest in‐patient developments in several key areas, focusing on mechanisms. We present advances in the use and action of several therapies including epigenetic modulators, protein degraders, apoptotic inducers, gene editing, and immunotherapy. We discuss the individual molecular mechanisms and clinical findings of these developments, emphasising their potential to improve survival and offer renewed hope for individuals with blood cancer. Importantly, such novel approaches with testing in patients should lead to further intensive efforts both at basic and translational research levels aiming at effective targeted and immunotherapy in blood cancers.

## 1 Introduction

The field of haematology has seen unprecedented development in biotechnology, significantly advancing the diagnosis and treatment of haematological malignancies and inherited blood disorders. Innovations including gene editing, immunotherapy, and the identification of novel small molecules have revolutionised patient outcomes and expanded therapeutic options, resulting in substantial patient benefits. Gene editing technologies such as CRISPR/Cas9 have enabled the ability to implement targeted changes to genetic material in a precise manner, offering potential cures for genetic disease such as sickle cell disease (SCD) and beta‐thalassaemia (BT) [1, 2]. Immunotherapeutic approaches, such as engineered monoclonal antibodies, bispecific T cell engagers, adoptive cell transfer with genetically engineered T cells that express chimeric antigen receptors (CAR), T cell receptors, and vaccines, have been promising in the treatment of haematologic malignancies, such as B cell lymphomas, acute myeloid leukaemia (AML), acute lymphoblastic leukaemia (ALL), and multiple myeloma (MM) [3, 4, 5]. Identification of small molecule therapies such as tyrosine kinase inhibitors (TKIs) and B cell lymphoma‐2 (Bcl‐2) inhibitors has enabled effective therapy in CML, AML, and systemic mastocytosis [6, 7, 8, 9, 10]. Advances in epigenetic modifications have added further to the therapeutic armamentarium with new possibilities for intervention in haematologic disorders [11, 12].

This review aims to provide a comprehensive overview of biotechnological advancements in the treatment of haematological malignancies in 2025, including their molecular mechanisms and clinical findings in detail. By evaluating the latest in‐patient studies and clinical trials, this work aims to provide insights into the innovative potential of these pioneering treatments on redefining the treatment horizon for haematologic malignancies (Table 1).

**TABLE 1:** List of all compounds discussed in this review.

Compound Name	Company	Disease(s)	Drug type	Clinical stage
OTX‐2002	Omega Therapeutics	HCC	Epigenomic controller	Phase I/II (NCT05497453)
KTX‐1001	K36 Therapeutics	r/rMM	MMSET inhibitor	Phase I (NCT05651932)
Revumenib	Syndax Pharmaceuticals	NPM1 r/rAML KMT2Ar AML KMT2Ar/ALL/MPAL	Menin inhibitor	Phase I/II (NCT04065399)
BN104	BioNova Pharmaceuticals	NPM1 r/rAML KMT2Ar AML KMT2Ar/ALL/MPAL	Menin inhibitor	Phase I/II study (NCT06052813)
Balamenib	Eilean Therapeutics	KMT2Ar AML	Menin inhibitor	Phase I (ACTRN12624000180516)
BGB‐16673	BeiGene	r/rCLL SLL	BTK protein degrader	Phase I/II (NCT05006716)
NX‐5948	Nurix Therapeutics	r/rCLL r/rSLL	BTK protein degrader	Phase Ia/b (NCT05131022)
KT‐253	Kymera Therapeutics	r/rAML ALL r/r Lymphoma Myelofibrosis r/r solid tumours	MDM2 protein degrader	Phase I (NCT05775406)
Sonrotoclax	BeiGene	r/rNHL FL DLBCL MZL t‐NHL MCL WM CLL SLL	Bcl‐2 inhibitor	Phase I (NCT04277637)
ABBV‐453	AbbVie	r/rMM r/rCLL r/rSLL	Bcl‐2 inhibitor	Phase I (NCT05308654) (NCT06291220)
Trem‐cel	Vor Biopharma	AML	CD33 gene therapy	Phase I/II (NCT04849910)
Vididencel	Mendus	AML Ovarian cancer	DC vaccine	Phase II (NCT03697707) Phase I (NCT04739527)
DOC1021	The Cooper Health System	GBM PDAC Angiosarcoma	DC vaccine	Phase I (NCT04552886)
AML‐VAC‐XS15	University Hospital Tuebingen	AML	Multi‐peptide vaccine	Phase I (NCT06252584)
CLN‐049	Cullinan Therapeutics Inc.	r/rAML MDS	[FLT3] × [CD3] bispecific antibody	Phase I (NCT05143996)
Glofitamab	Hoffmann‐La Roche	B‐NHL	[CD20] × [CD3] bispecific antibody	Phase I (NCT03075696)
Epcoritamab	Genmab	r/rCLL r/rSLL RS	[CD20] × [CD3] bispecific antibody	Phase Ib/II (NCT04623541)
ICT01	ImCheck Therapeutics	Bladder cancer Breast cancer Colon cancer Gastric cancer Ovarian cancer Prostate cancer elanoma PDAC AML FL HNSCC	Monoclonal antibody targeting BTN3A	Phase I (NCT04243499)
INCA033989	Incyte Corporation	MPN	Monoclonal antibody targeting mutant CALR	Phase I (NCT06034002) (NCT05936359)
JNJ‐88549968	Janssen Research & Development LLC	MPN	[mutant CALR] × [CD3] bispecific antibody	Phase I (NCT06150157)
MYTX‐011	Mythic Therapeutics	NSCLC	ADC targeting c‐MET	Phase I (NCT05652868)

Abbreviations: ADC, antibody‐drug conjugate; ALL, acute lymphoblastic leukaemia; AML, acute myeloid leukaemia; Bcl‐2, B cell lymphoma‐2; BTK, Bruton's tyrosine kinase; BTN3A, butyrophilin 3A; CALR, calreticulin; CLL, chronic lymphocytic leukaemia; c‐MET, mesenchymal‐epithelial transition factor; DC, dendritic cell; FL, follicular lymphoma; GBM, glioblastoma; HCC, hepatocellular carcinoma; HNSCC, head and neck squamous cell carcinoma; KMT2Ar, histone‐lysine N‐methyltransferase 2A rearranged; MCL, mantle cell lymphoma; MDM2, mouse double minute 2 homologue; MDS, myelodysplastic syndrome; MM, multiple myeloma; MMSET, multiple myeloma SET domain‐containing protein; MPAL, mixed‐phenotype acute leukaemia; MPN, myeloproliferative neoplasms; MZL, marginal zone lymphoma; NHL, non‐Hodgkin lymphoma; NPM1, nucleophosmin 1; NSCLC, non‐small cell lung cancer; PDAC, pancreatic ductal adenocarcinoma; r/r, relapsed/refractory; RS, Richter's syndrome; SLL, small lymphocytic lymphoma; t‐NHL, transformed NHL; WM, Waldenströms macroglobulinaemia.

## 2 Epigenetic Modulators

Epigenetics encompasses a range of biological processes that regulate DNA without altering its nucleotide sequence [13]. Epigenetic modifications such as DNA methylation and histone modification are carried out by protein complexes categorised into writers, readers, and erasers. The epigenetic writers introduce specific chemical modifications to DNA or histones, creating epigenetic marks. These include DNA methyltransferases (DNMTs), such as DNMT1, histone acetyltransferases (HATs) such as CREB binding protein (CBP)/p300 and histone methyltransferases (HMTs), which include enhancer of zeste homologue 2 (EZH2) [14]. Readers, which include methyl‐CpG‐binding domain proteins (MBPs) and bromodomain (BRD)‐containing proteins, recognise and interpret these modified protein domains, whereas enzymes such as histone deacetylases (HDACs), demethylases, deubiquitinases, and ten‐eleven translocation (TET) proteins act as erasers, removing these epigenetic marks [14]. Groundbreaking research over the past decades has highlighted epigenetic regulation as a crucial factor in tumorigenesis. Significant findings have shown that variations in epigenetic control, specific to cell type and stage of differentiation, dictate the timing and location of cellular transformation [15, 16, 17]. Indeed, dysregulation of epigenetic processes is a known driver of haematologic disorders [18].

The hypomethylating agents (HMAs) azacitidine and decitabine have significantly improved survival and response rates in patients with AML and myelodysplastic syndrome (MDS), in particular, in those who are unfit for traditional chemotherapy [19, 20]. Despite their statistical significance regarding overall response rates, it remains that at least 60%‐70% of patients treated with azacitidine and over 70% of patients treated with decitabine do not effectively respond [21, 22]. In addition to severe side effects such as neutropenia in up to 66% of patients, azacitidine treatment requires frequent daily injections, thus significantly impacting patient quality of life [23, 24]. Consequently, there is an ongoing and critical need to further enhance and refine epigenetic therapies.

### 2.1 OTX‐2002

*Mechanism: Epigenetic control of gene expression through insulated genomic domains*.

*Disease: HCC*.

**FIGURE 1:**
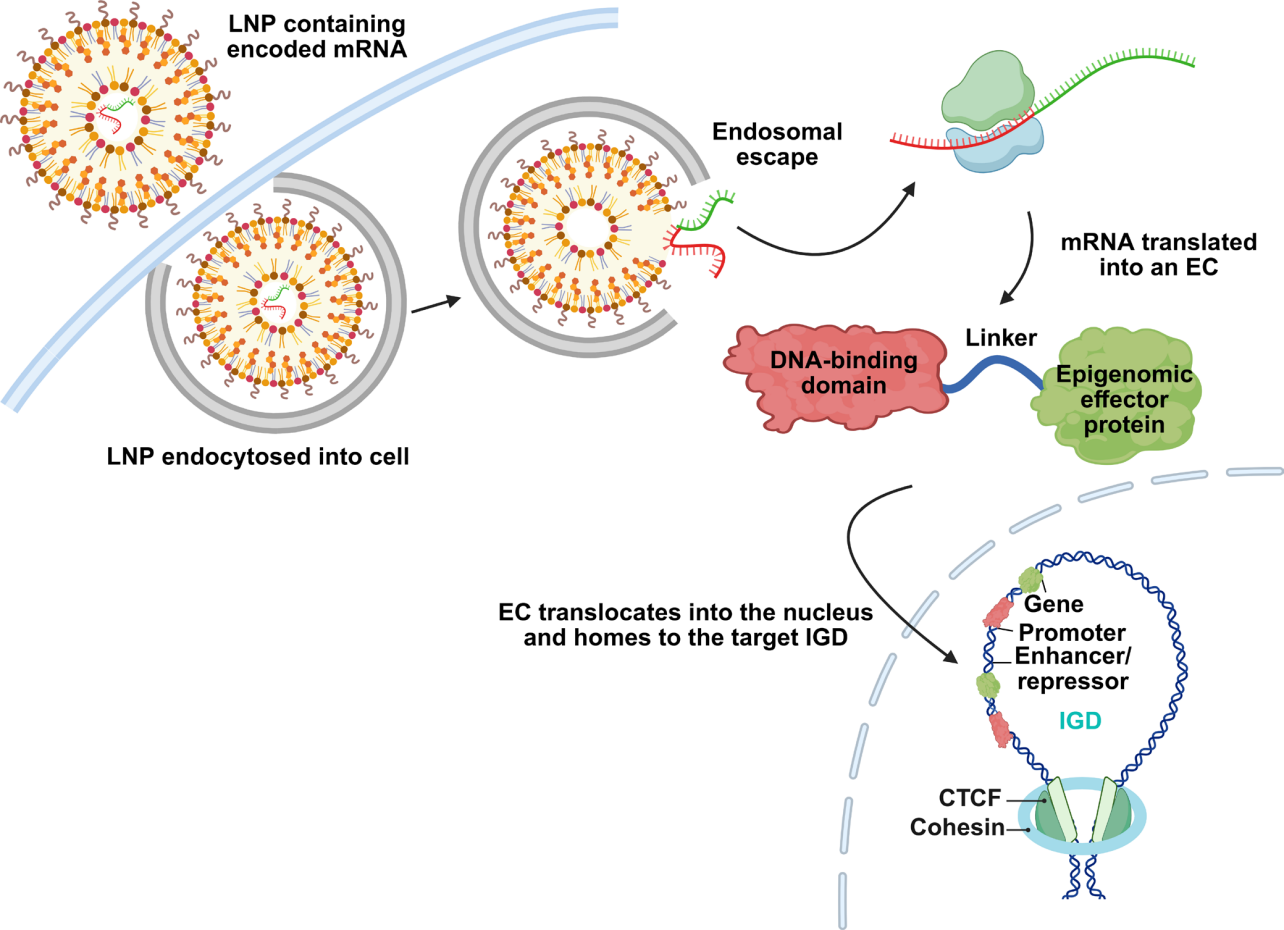
Mechanism of action of Epigenomic Controllers. The mRNA encoding the ECs are encapsulated in LNPs and enter the cell through endocytosis. Following endosomal escape, the mRNA is translated to produce a fusion protein, the EC. The EC contains a specific DNA‐binding domain and an epigenomic effector, that is capable of regulating gene expression through harnessing the various forms of epigenetic modifications, including DNA methylation and acetylation of histones. Once translated, the EC translocates into the nucleus and homes into the target IGD. At the IGD, the EC will perform the desired epigenetic modification at a specific sequence, known as the ‘Epigenomic Zipcode.’ LNP, lipid nanoparticle; mRNA, messenger RNA; EC, Epigenomic Controller; IGD, insulated genomic domain; CTCF, CCCTC‐binding factor.

One original and promising strategy involves leveraging the innovative technology of Epigenomic Controllers (ECs) (Figure 1) to precisely regulate gene expression by targeting insulated genomic domains (IGDs), also called Insulated Neighbourhoods [25]. An EC is a synthetic messenger RNA (mRNA), formulated in lipid nanoparticles (LNPs), that is translated inside the target cell into a programmable DNA‐binding protein fused to an epigenetic modifier such as DNMTs, HDACs, or polycomb‐repressive‐complex components (PRCs). After translation, the fusion protein binds a predefined DNA sequence within the specific IGD and subsequently recruits its epigenetic effector. This locus‐selective recruitment permits a precise, tunable, and durable approach to gene regulation. The average size of IGDs is 186 Kb, which are formed by chromosomal loops anchored by CCCTC‐binding factor (CTCF) and the cohesin complex. They act as topological safeguards, ensuring that enhancers and promoters are maintained within the same loop and prevent unintended interactions with neighbouring genes. Guiding epigenetic modifiers to a single IGD therefore allows tightly controlled repression or activation of its resident gene(s) while sparing the rest of the genome [26].

The first‐in‐class EC, OTX‐2002 has demonstrated significant anti‐tumour responses in pre‐clinical testing and promising safety and pharmacokinetic profiles in a Phase I/II clinical trial (NCT05497453) with 89% of the majority of treatment‐related adverse events (TRAEs) being low grade (89%), with no dose‐limiting toxicities (DLTs) at doses up to 0.12 mg/kg. The OTX‐2002 EC is a bicistronic mRNA that is encapsulated in LNPs, engineered to express two proteins which work together to epigenetically silence MYC expression by acting on its IGD. The first protein includes a zinc finger (ZF) DNA‐binding domain fused to a DNMT (MQ1), which methylates CpG sites near the MYC promoter inhibiting access to transcription factors, designated as MYC‐EC‐E1. The other protein, MYC‐EC‐E2, includes a ZF DNA‐binding domain targeting a MYC enhancer fused to a Krüppel‐associated box (KRAB) domain, which deposes H3K9me3 marks resulting in the formation of heterochromatin [25]. Thus, this dual targeting of the MYC IGD creates a genomic environment which isolates MYC from external activation. While the specificity is given by the ZF binding domains targeting the promoter and enhancer, it will be important to assess off‐targets effects as those DNA binding domains have also other targets than MYC regulatory regions.

Evidence based on a hepatocellular carcinoma (HCC) model indicates that not only is there sufficient methylation of the MYC IGD resulting in its targeted inhibition and cell death, but also that these methylation marks are maintained for up to 15 days post‐treatment indicative of long‐lasting repression. In comparison to other gene modifying techniques such as CRISPR‐based methods, OTX‐2002 lacks any additional components such as guide RNAs (gRNAs), thereby simplifying the delivery process. Additionally, the epigenetic silencing of genes could be an avenue to bypass these resistance mechanisms commonly associated with small molecule inhibitors. This technology is also being harnessed to potentially treat β‐ and α‐thalassaemia by leveraging the multi‐gene control capabilities of ECs to modulate expression of haemoglobin subunit alpha 1 (HBA1) and haemoglobin subunit alpha 2 (HBA2) at specific cis‐regulatory elements (cREs) within their IGDs [27]. A total of 15,000 unique IGDs have been identified, suggesting that virtually any gene could potentially be targeted [28]. Indeed, the future advancement of this technology holds great promise, particularly in its potential to treat leukaemias with genetic and karyotypic profiles associated with poor prognosis and that have historically been difficult to manage. It should be noted that Omega Therapeutics recently announced the termination of their OTX‐2002 clinical trial, though detailed reasons for the discontinuation have not yet been disclosed.

### 2.2 KTX‐1001

*Mechanism: inhibition of MMSET by binding to the SAM‐binding pocket within the SET domain*.

*Disease: MM*.

Multiple myeloma SET domain‐containing protein (MMSET) is a histone lysine methyltransferase that is involved in regulating chromatin structure and gene expression. It catalyses the methylation of histone H3 at lysine 36 (H3K36), which is associated with active transcription and chromatin re‐modelling. Dysregulation of MMSET through chromosomal translocations, mutations, or overexpression is a hallmark of several cancers, particularly the t(4;14) translocation, which occurs in 10%‐15% of MM and leads to MMSET overexpression, an established contributor to chemoresistance and poor prognosis [29]. KTX‐1001, a first‐in‐class, selective small molecule inhibitor, is designed to specifically target and inhibit MMSET activity. By binding with high affinity to the s‐adenosyl methionine (SAM)‐binding pocket within the SET domain, KTX‐1001 blocks the histone methyltransferase activity of MMSET and avoids SAM competition. Encouraging results from a Phase I clinical trial (NCT05651932) in patients with relapsed and refractory MM (r/rMM) showed that none of the treated patients experienced dose‐limiting toxicities (DLTs), with good tolerability overall. Notably, two heavily pre‐treated patients displayed long‐lasting stable disease. Assessment of pharmacokinetics and pharmacodynamics is indicative of a H3K36me2 targeted mechanism. Although these results are promising, it is important to consider the potential for acquired resistance through mutations of the SET domain.

### 2.3 Revumenib

*Mechanism: menin inhibitor, prevents KMT2A from acting on chromatin*.

*Disease: AML, ALL, MPAL*.

The highly aggressive AML with histone‐lysine N‐methyltransferase 2A rearrangements (KMT2Ar) presents with poor prognosis and a median overall survival of just 2.4 months [30]. Genetic rearrangements of KMT2A result in oncogenic overexpression of homeobox (HOX) genes and their DNA‐binding cofactor MEIS1 overall inducing a block in differentiation and initiation of leukemogenesis through aberrant epigenetic changes. Menin serves as a key oncogenic cofactor that drives these leukaemic processes, and all known KMT2A fusion proteins retain the menin‐binding motif which is required for binding and activating HOX gene promoters [31]. Menin is also required for the oncogenic effect of nucleophosmin 1 (NPM1) mutants, as they lead to aberrant HOX and MEIS1 expression [32]. Thus, patients with leukaemia harbouring the KMT2Ar and NPM1 mutations may benefit from being treated with menin inhibitors.

The first Food and Drug Administration (FDA)‐approved menin inhibitor, revumenib (SNDX‐5613) has demonstrated encouraging clinical results, achieving an overall response rate (ORR) of 63%, with 23% of patients achieving complete remission (CR) or CR with partial haematologic recovery (CRh) (NCT04065399). Additionally, 70% of patients with CR + CRh achieved measurable residual disease (MRD) negativity. Despite these encouraging results, TRAEs were observed in 82% of patients and QTc prolongation occurring in 23% of patients. Furthermore, the overproduction of chemokines in the lungs which promotes a hyperinflammatory state in response to anti‐leukaemic therapy, known as differentiation syndrome (DS), was present in 27% of patients. Mechanistically, revumenib occupies the KMT2A‐binding pocket on menin, forming crucial hydrophobic and hydrogen‐bond contacts with residues W346, M327 and G331. This competitive binding disrupts the menin‐KMT2A chromatin complex, down‐regulates HOXA and MEIS1, and permits myeloid differentiation. Furthermore, patients rapidly accumulated MEN1 mutations within two cycles of treatment in key residues M327 and G331. These mutations lead to steric hindrance with the target residue W346, which forms part of the hydrophobic core that is essential for revumenib stabilisation with menin [33].

### 2.4 BN104

*Mechanism: menin inhibitor, prevents KMT2A from acting on chromatin*.

*Disease: AML, ALL*.

BN104, a third selective non‐covalent menin inhibitor, designed to maintain high pocket affinity while reducing dependence on the W346 hydrogen bond, has demonstrated promising results in preclinical and early‐phase clinical studies. Minimising reliance on W346 may mitigate the emergence of resistance mutations, as seen with first‐generation menin inhibitors like revumenib, which rely heavily on this residue for stable binding. The FDA has granted it orphan drug designation for the treatment of AML. Initial data from a Phase I/II study (NCT06052813) in relapsed/refractory AML (r/rAML) patients demonstrated a promising safety profile and significant antileukemic activity, with an ORR of 89% and 33% achieving CR. Important molecular targets such as HOXA and MEIS1 were downregulated whereas genes implicated in differentiation such as CD11b were upregulated. However, 90% of patients experienced TRAEs, with 60% of patients experiencing grade 3 or above [34].

### 2.5 Balamenib

*Mechanism: menin inhibitor, prevents KMT2A from acting on chromatin*.

*Disease: AML*.

To circumvent the diminished therapeutic effect and cardiac toxicity associated with revumenib and the high percentages of TRAEs with BN104, the second‐generation menin inhibitor balamenib (ZE63‐0302), could be an effective therapeutic for clinicians to treat KMT2Ar AML. Balamenib confers a unique binding modality which permits an energetically favourable conformation allowing the ligand to exhibit target interaction whilst also avoiding W346, reducing the emergence of the aforementioned resistance mutations. The initial results from a Phase I trial (ACTRN12624000180516) of healthy volunteers demonstrated no significant cardiotoxicity allowing for safe administration at higher doses without the concerns of QTc prolongation that was observed in revumenib. It is yet to be recognised whether these positive effects of balamenib would be achieved in AML patients [35].

Altogether, these inhibitors demonstrate the emphasis that drug development has towards overcoming early resistance challenges. Revumenib established proof of concept for menin inhibition in KMT2Ar AML, though its clinical utility has been constrained by resistance mutations arising from reliance on W346‐mediated binding. In response, BN104 and balamenib were rationally engineered to circumvent this structural vulnerability, with the aim of improving clinical response whilst enhancing resistance profiles and therapeutic durability.

## 3 Protein Degraders

Targeting the B cell receptor (BCR) signalling pathway, particularly through inhibiting Bruton's tyrosine kinase (BTK), has dramatically transformed the lives of patients with CLL. While BTK inhibitors (BTKis) such as ibrutinib have significantly improved patient outcome, the associated platelet and cardiac myocyte toxicity, in addition to the emergence of resistance mutations within the tyrosine kinase catalytic domain, remain a substantial challenge. These toxicities are suggested to be due in part to the inhibition of three major kinases, epidermal growth factor receptor (EGFR), interleukin‐2 inducible kinase (ITK) and the tyrosine kinase 3 (TEC) family of kinases [36]. A novel class of BTK‐targeting drugs, known as BTK degraders, offers a potential solution.

### 3.1 BGB‐16673

***Mechanism**:* induces BTK degradation via polyubiquitination.

*Disease: CLL, SLL*.

BGB‐16673 is a chimeric heterobifunctional degradation inducing small molecule that is comprised of a BTK‐binding moiety conjugated to a linker and a cereblon (CRBN) E3 ligase binder that induces BTK degradation via polyubiquitination. In preclinical models, BGB‐16673 effectively degraded both wild‐type BTK and various BTK mutant forms resistant to covalent and noncovalent BTK inhibitors (BTKi), resulting in significant tumour suppression [37]. The FDA has granted fast track designation to BGB‐16673 for adult patients with relapsed or refractory CLL (r/rCLL) or small lymphocytic lymphoma (SLL) who have previously received two or more prior lines of therapy [38]. The designation is based on data from a Phase I/II clinical trial (NCT05006716). At a median follow‐up of 4.6 months, the ORR was 72%. The disease control rate (DCR) and median time to first response were 88% and 2.8 months, respectively. Importantly, positive responses were recorded in patients with del(17p) or *TP*53 mutation (68%) and those which presented with the BTK resistance mutations C481S, T474l, and/or L528S. A Phase II cohort of patients with CLL/SLL exposed to both a BTK inhibitor and Bcl‐2 inhibitor is now enrolling [38].

### 3.2 NX‐5948

***Mechanism**:* induces BTK degradation via polyubiquitination.

*Disease: CLL, SLL*.

Another BTK‐targeted degrader, NX‐5948, which is an advancement of its predecessor NX‐2127, has also demonstrated rapid, robust and sustained BTK degradation. This novel oral small molecule induces BTK degradation in a similar mechanism as BGB‐16673 via recruitment of the CRBN E3 ubiquitin ligase complex. NX‐2127 has achieved subnanomolar potency degradation of both wild‐type and common mutant forms of BTK, with degradation occurring within 2 h of oral administration [39]. A feature stemming from its molecular design that separates NX‐5948 from other BTK‐targeted proteolysis‐targeting chimeras (PROTACs) is its ability to cross the blood–brain barrier and degrade BTK intracranially, translating to preclinical efficacy in CNS lymphomas [40]. NX‐5948 is currently being assessed in a Phase Ia/b trial in patients with r/rCLL, that currently has enrolled 87 patients (NCT05131022). NX‐5948 demonstrated a promising safety and efficacy profile. In 30 evaluable patients, the ORR was 77%. Common adverse events included purpura (44.1%) and thrombocytopenia (23.5%). No new‐onset atrial fibrillation or hypertension was reported. Responses were observed in challenging subgroups, including those with prior Bcl‐2i/BTKi exposure, *TP*53 and BTK mutations. Longer treatment duration correlated with improved outcomes, as 5 patients converted from stable disease to partial response. These treatment responses resulted in NX‐5948 being granted fast track status by the FDA to treat r/rCLL [41].

Despite the promising clinical findings of E3‐based BTK degraders, emerging studies are beginning to unveil that CLL patients can acquire resistance to these novel drugs. For instance, a CLL patient was treated with BGB‐16673 after developing progressive disease following previous treatments that included venetoclax and ibrutinib. After a significant initial reduction in bulky lymphadenopathy, the patient developed resistance to BGB‐16673, leading to rapidly progressive disease and resistance to all available therapies. It was revealed that the A428D mutation, located within the ATP‐binding interface of BTK, was responsible for the resistance. This mutation impairs the autophosphorylation of BTK and downstream signalling but enables enhanced calcium flux and increases cell survival by activating AKT, extracellular signal‐regulated kinase (ERK), and nuclear factor‐κB (NF‐κB). These findings raise important concerns regarding the long‐term efficacy of BTK‐targeted approaches and highlight that these novel small molecules are not resistant to acquired mutations [42].

### 3.3 KT‐253

***Mechanism**: induces MDM2 degradation* via *polyubiquitination*.

**Disease:** AML, ALL, lymphoma, myelofibrosis.

A primary mechanism that is responsible for disrupting the function of wild‐type p53 is proteasomal degradation, which is initiated by ubiquitination and catalysed by the E3 ubiquitin ligase murine double minute 2 (MDM2) [43]. Due to the crucial tumour suppressor function of p53 in cancer, there is a compelling rationale for inactivating MDM2 to enhance the activity and stability of wild‐type p53. KT‐253 is a heterobifunctional molecule consisting of a potent MDM2 ligand connected through a linker to a high‐affinity ligand for CRBN, allowing for subnanomolar degradation and stabilisation of p53. In vivo studies have demonstrated rapid apoptosis and sustained tumour regression in wild‐type p53 AML and ALL. A preclinical myelofibrosis (MF) model demonstrated significant inhibition of cell growth and increased apoptosis in MF CD34^+^ cells, which was > 10‐fold higher than when treated with the MDM2 inhibitor SMI AMG‐232. There was also selective death of JAK2 V617F^+^ colony forming MF cells without affecting normal cells [44]. KT‐253 has progressed into an ongoing Phase I clinical trial (NCT05775406) which includes patients with AML, ALL, lymphoma, and solid tumours. To date, over 20% of patients' have demonstrated common adverse events (AEs), including nausea, fatigue, headache. There has been one patient that has achieved CR and one patient who has demonstrated a partial response (PR) within the AML arm. Overall, KT‐253 has activated p53‐dependent signalling in patients, which appears to be responsible for its anti‐cancer effects, at a dose that was well‐tolerated. However, this approach requires caution due to the potential risk of selecting for or inducing *TP*53 mutations.

## 4 Bcl‐2 Inhibitors

Venetoclax has revolutionised the treatment landscape for CLL and AML. Its selective binding to the Bcl‐2 protein and subsequent cellular apoptosis has led to significant improvements in patient outcomes, including higher response rates and prolonged survival. However, challenges remain, such as acquired resistance and the need to reduce toxicity [45].

### 4.1 Sonrotoclax

*Mechanism: Bcl‐2 inhibitor that overcomes the Bcl‐2 G101V that is associated with venetoclax resistance*.

*Disease: NHL, FL, DLBCL, MZL, MCL, WM, CLL, SLL*.

Multiple studies have highlighted the frequency of the Bcl‐2 G101V mutation in patients with r/rCLL who have been treated with venetoclax. This mutation prevents the binding of venetoclax to Bcl‐2, thereby giving rise to drug resistance [46]. Greater potency and specificity have been demonstrated with the second‐generation Bcl‐2 inhibitor sonrotoclax compared with venetoclax. Preclinical results utilising cancer cell lines and mouse models have demonstrated that sonrotoclax is effective against both wild‐type Bcl‐2 and Bcl‐2 G101V, suggesting its potential to overcome venetoclax resistance associated with this mutation. The molecular mechanism responsible for the Bcl‐2 G101V mutation involves a rotamer change in the E152 residue with subsequent repositioning of venetoclax, therefore reducing the binding affinity of the compound. However, upon binding of Bcl‐2 G101V with sonrotoclax, the mutation is too distant from sonrotoclax resulting in minimal effect on binding interactions even through E152. Hence, the binding of sonrotoclax to Bcl‐2 is less susceptible to the downstream effects of the G101V mutation [47]. Results from the current Phase I clinical trial (NCT04277637) comparing the responses to sonrotoclax monotherapy and in combination with the BTKi zanubrutinib for the treatment of patients with r/rCLL/SLL have demonstrated an ORR of 97% and high undetectable minimal residual disease (uMRD) rates. Additionally, the combination therapy demonstrated an acceptable tolerability profile for all administered regimens, with only one patient discontinuing due to disease progression [48].

### 4.2 ABBV‐453

*Mechanism: Bcl‐2 inhibitor*.

*Disease: MM, CLL, SLL*.

The next‐generation Bcl‐2 inhibitor, ABBV‐453, was engineered for optimal molecular and biological properties, with the aim of improving clinical outcomes compared with existing therapies. By incorporating macrocyclic elements into the hot‐spot binding sites of Bcl‐2, ABBV‐453 presents as a constrained molecule with enhanced potency, selectivity, and extended in vivo activity at low doses. In vitro studies have demonstrated that ABBV‐453 had subnanomolar binding to Bcl‐2, with greater selectivity for Bcl‐2 than for all other members of the Bcl‐2 family, such as B cell lymphoma‐extra large (Bcl‐xL) and myeloid cell leukaemia‐1 (MCL‐1). Furthermore, ABBV‐453 was also found to exhibit high cytotoxic activity in cell lines that were dependent on Bcl‐2, MCL‐1, or Bcl‐xL for survival. This cellular response was due to disruption of the Bcl‐2:Bim complexes and subsequent activation of the intrinsic apoptotic pathway. In vivo studies of ALL demonstrated that at equivalent doses and schedules, ABBV‐453 as a monotherapy was significantly more effective in inhibiting tumour growth than other clinically relevant Bcl‐2 inhibitors, including sonrotoclax and lisaftoclax [49]. As a result of these findings, ABBV‐453 is actively being investigated in a Phase I clinical trial in patients with r/rMM (NCT05308654) and r/rCLL/SLL (NCT06291220).

## 5 Gene Therapy

Recent breakthroughs in gene therapy such as exagamglogene autotemcel (Exa‐cel, Casgevy) and betibeglogene autotemcel (beti‐cel, Zynteglo) have revolutionised the treatment of SCD and β‐thalassaemia by correcting the effects of causal genetic mutations. A single infusion of genetically modified cells could offer lasting therapeutic effects and potentially cure both conditions [50, 51]. However, there is a potential risk of the development of MDS or AML in SCD due to inflammation, which impairs haematopoiesis [52].

Despite allogeneic haematopoietic cell transplantation (HCT) being the standard of care for most AML patients at high risk of relapse, relapse‐free and overall survival for many patients remains poor, highlighting an urgent need to improve HCT outcomes [53]. CAR‐T therapy has demonstrated unprecedented success against B cell malignancies, offering curative outcomes for certain patients [54]. However, the clinical efficacy of CAR‐T therapy is hampered by the requirement for cell surface makers that are exclusively or preferentially expressed on cancer cells to minimise toxicity against healthy cells, known as “on‐target, off‐tumour” toxicity. Indeed, most AML antigens, like CD33, are also found on normal myeloid cells, including HSCs, limiting the use of antigen‐directed therapies [55]. This issue has hampered the long‐term use of CD33 directed therapies such as gemtuzumab ozogamicin (GO) due to the severe myelotoxicity caused by the on‐target, off‐tumour activity.

### 5.1 Tremtelectogene Empogeditemcel

*Mechanism: CRISPR‐Cas9 based gene therapy to delete CD33 on HSPCs*.

*Disease: AML*.

To eliminate on‐target toxicity of CD33‐directed therapies, tremtelectogene empogeditemcel (trem‐cel) has been developed. Trem‐cel is an allogeneic, human leukocyte antigen (HLA)‐matched, CRISPR‐Cas9‐edited product to be administrated to HCT‐eligible AML patients who are at high risk for relapse and post‐transplant mortality. Healthy haematopoietic stem and progenitor cells (HSPCs) were attained from human donors and electroporated with a pre‐complexed ribonucleoprotein (RNP) consisting of a CRISPR gRNA targeting the common exon 3 of CD33 combined with a Cas9 protein which resulted in > 70% gene editing efficiency. In vivo studies were indicative that trem‐cel, manufactured at clinical scale, is maintained long‐term in haematopoietic tissue and facilitates effective multilineage repopulation and ensures that CD33‐null HSPCs function appropriately. All engrafted mice demonstrated persistent CD33 editing within the bone marrow 16 weeks following transplant, with the most frequent indels (1‐bp and 2‐bp deletions) not providing a positive nor negative selective advantage [56]. Most importantly, all edited mice were resistant to GO treatment.

As a result of these finding a Phase I/II trial (NCT04849910) has been initiated to establish the safety of using trem‐cel as an allograft followed by GO maintenance therapy for patients with CD33^+^ AML or MDS who are at high risk of relapse and undergoing HCT. The initial results are encouraging, with trem‐cel demonstrating rapid engraftment and sustained haematopoiesis, alongside consistent depletion of CD33^+^ myeloid cells. Additionally, no prolonged cytopenias that are commonly associated with GO were observed in the CD33‐deleted haematopoietic cells. With regard to pharmacokinetics, trem‐cel patients experienced similar effective drug exposure levels as the higher doses used in r/rAML patients, that indicates that trem‐cel may permit lower dosing of GO while still achieving effective exposure [57]. The development of trem‐cel, the first of its kind, highlights a therapeutic framework for identifying other dispensable gene targets that could be edited to improve patient responses to immunotherapy while minimising associated toxicities.

## 6 Cancer Vaccines

Ever since the Bacillus Calmette‐Guérin (BCG) vaccine became the first immunotherapy of any type to receive FDA approval in 1990, there have been significant efforts in the oncology field to implement vaccines to target other cancers [58]. Dendritic cells (DCs) are professional antigen‐presenting cells (APCs) that are crucial in bridging innate and adaptive immunity. They play essential roles in triggering specific cellular and humoral responses against tumour and infectious antigens. DCs are unique in their ability to interact with naive T cells, inducing their activation, differentiation, and effector responses. Additionally, their fundamental role in maintaining immunological tolerance positions them as an attractive target for several therapeutic approaches, including anti‐cancer therapies [59]. In solid cancers and leukaemia, the capability of DCs to present tumour antigens to T cells, in particular the priming of CD8^+^ cytotoxic T‐lymphocytes (CTLs), has been harnessed to develop DC‐based vaccines. DC vaccines can be broadly categorised into either being allogenic or autologous. Allogeneic DC vaccines use donor‐derived DCs, resulting in a broad antigen repertoire and potent immune responses through polyclonal T cell activation. Contrastingly, autologous DC vaccines utilise the patient's own cells, which provide a more tailored treatment but are associated with challenges in scalability, cost, and limited antigen presentation due to tumour heterogeneity [60].

### 6.1 Vididencel

***Mechanism**:* *allogeneic DC vaccine harbouring AML‐associated antigens*.

*Disease: AML, ovarian cancer*.

Vididencel is an allogeneic DC vaccine to be injected intradermally, that uniquely combines the anti‐cancer properties of allogenic DC vaccines and multi‐antigen‐expressing tumour cell vaccines. The vaccine contains mature DCs that were produced from differentiating the AML cell line, DCOne, thus it also harbours AML‐associated antigens. Additionally, the cells express high levels of CD1a, langerin, several costimulatory molecules, and major histocompatibility complex (MHC) class I/II molecules. Allogeneic T cells were consistently primed and would migrate following stimulation by chemokines C‐C motif chemokine ligand 21 (CCL21) and C‐C motif chemokine ligand 19 (CCL19) in vitro [61]. The current Phase II clinical trial (NCT03697707) treating AML patients with vididencel has demonstrated safe and feasible results, with side effects restricted to reactions at the local injection site. Patients receiving vididencel as monotherapy following intensive induction therapy illustrated strong T cell responses and durable disease control. The greatest clinical benefit was observed in patients who displayed a sustained T cell response post‐treatment, along with an immune‐competent profile characterised by a high percentage of B cells and minimal immune‐suppressive T cells (CD8^+^LAG‐3^+^ central memory T cells). The estimated 1‐and 3‐year relapse‐free (RFS) is 75% and 57%, respectively. The estimated 1‐and 3‐year OS is 88% and 69%, respectively. Emerging in vivo work has shown synergism when vididencel was combined with azacitidine and venetoclax, improving tumour growth inhibition [62].

### 6.2 DOC1021

***Mechanism**: **patient**‐derived DC vaccine that express antigens that are recognised by both MHC class I and II molecules, resulting in activation of* *CD8* ^+^ *cytotoxic T cells and CD4* ^+^ *helper T cells.*

*Disease: GBM, PDAC, Angiosarcoma*.

**FIGURE 2:**
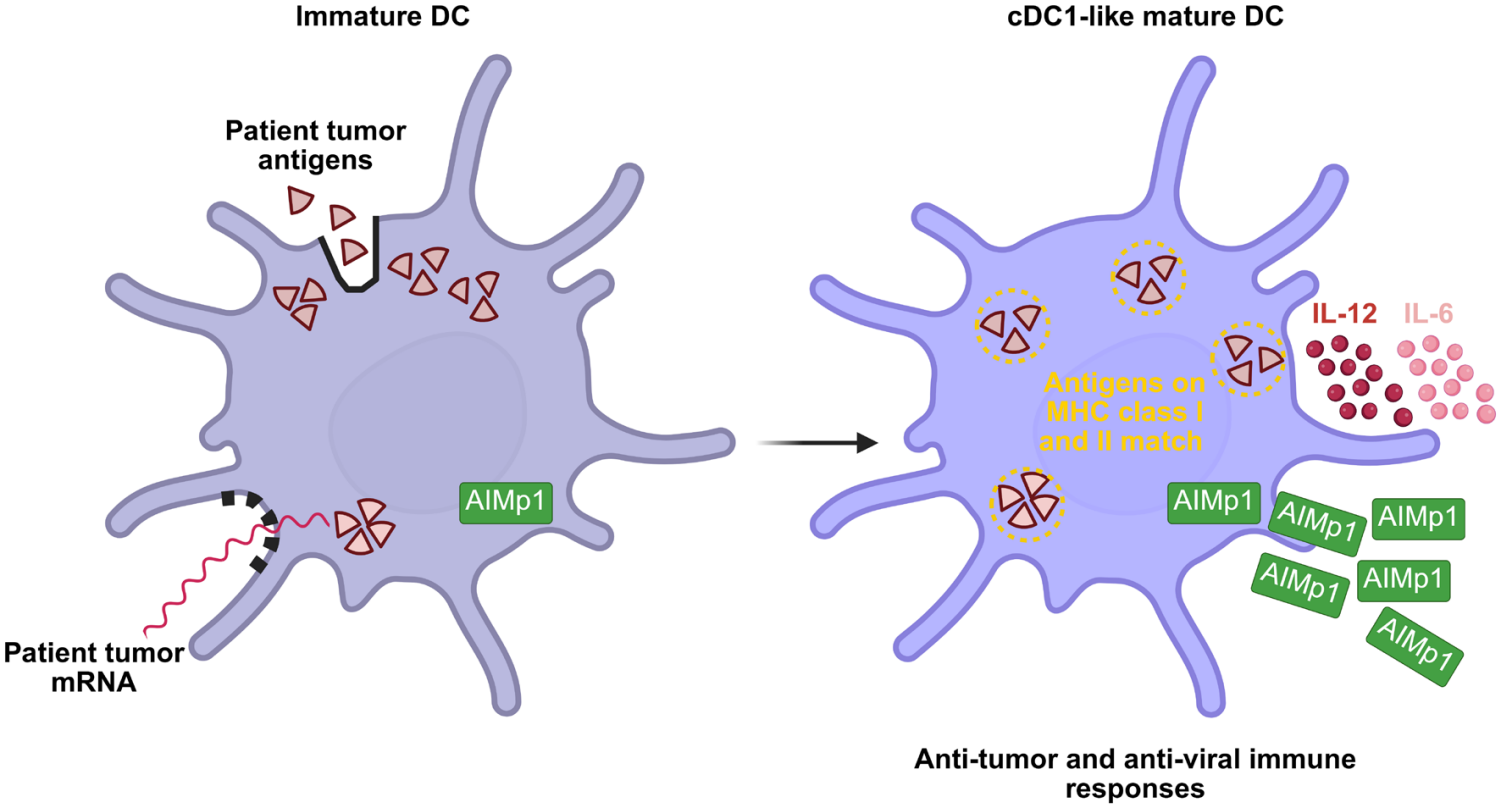
DOC1021 mechanism of action. The vaccine consists of incorporating patient DCs with tumour antigens and mRNA, both internally and externally, known as ‘double‐loading’ that stimulates a viral infection. The matched antigens on MHC class I and II initiate a cascade of events including DC Th1 polarisation and subsequent enhancement of CD8^+^ responses, mediated by the release of AIMp1 and pro‐inflammatory and immunoregulatory cytokines. Ultimately, reprogramming DCs to adopt a cDC1‐like phenotype. mRNA, messenger RNA; AIMp1, aminoacyl tRNA synthetase‐interacting multifunctional protein 1; MHC class I, major histocompatibility complex I; MHC class II, major histocompatibility complex II; IL‐12, interleukin‐12; IL‐6, interleukin‐6; DC, dendritic cell; cDC1, conventional type 1 DC.

Another emerging platform that harnesses the anti‐tumour potential of DC vaccines would be DOC1021 (Figure 2). This is a cell‐based vaccine that employs homologous antigenic loading, also known as ‘double‐loading,’ that enables multiple tumour‐associated antigens to be targeted simultaneously. In essence, this double‐loading involves incorporating DCs with tumour‐specific antigens both internally and externally. Internally, the DCs are loaded with RNA from the tumour, ensuring antigen presentation on MHC class I molecules. Externally, the same antigens are loaded with proteins from the tumour onto the DCs to mimic a viral infection, leading to presentation on MHC class II molecules. This dual presentation activates both CD8^+^ cytotoxic T cells and CD4^+^ helper T cells, resulting in a robust and targeted immune response against the cancer cells. The process begins by harvesting patient tumour tissue and extracting the protein and RNA. Apheresis is conducted on the patient to which the DCs are then isolated and double‐loaded. MHC class I antigens are delivered to these DCs through electroporation of amplified autologous tumour mRNA whereas MHC class II antigens are delivered by subsequent DC incubation with autologous tumour lysate. The vaccine is administered to the patient through ultrasound‐guided injections into the cervical lymph nodes, with a schedule of three doses every 2 weeks, concurrently supplemented with weekly interferon‐alpha (IFN‐α) injections [63].

The homologous antigenic loading is recognised by the innate immune system as a pathogen‐associated molecular pattern (PAMP). This results in the activation of the aminoacyl tRNA synthetase‐interacting multifunctional protein 1 (AIMp1). AIMp1 is a secreted inflammatory cytokine, and its expression in DCs has been shown to play an essential role in Th1 polarisation, interleukin‐6 (IL‐6) and interleukin‐12 (IL‐12) production, overall inducing DC maturation. AIMp1 has also been shown to stimulate monocytes/macrophages through inducing phosphorylation of p38 mitogen‐activated protein kinase (MAPK) and inhibitor of nuclear factor kappa‐B kinase subunit alpha/beta (IKKα/β), resulting in the nuclear translocation of p65 and activation of NF‐κB signalling. Following the activation of AIMp1 by DOC1021, the p38 MAPK and mammalian target of rapamycin complex 1 (mTORC1) cascades are subsequently activated, ultimately reprogramming the DCs to adopt a conventional type 1 DC (cDC1)‐like phenotype [64]. This phenotype enhances the ability of DCs to prime CD8^+^ T cells with improved cytotoxic activity, memory formation, and resistance to exhaustion. Notably, unlike CAR‐T cell therapy, DOC1021 does not require genetic modification or myeloablative chemotherapy. Additionally, the “double loading” of DCs generates a 10‐fold stronger activation of cytotoxic T cells, when compared to DCs loaded with only tumour protein or mRNA. Histological and flow cytometric analyses conducted after patient treatment, confirmed the presence of activated CD8+ T cells and enhanced immune responses in tumour tissues, demonstrated by increased circulating CD8^+^ and CD4^+^ central memory T cells and CD8^+^ memory precursor effector cells (MPECs).

Based on the current findings from the Phase I clinical trial (NCT04552886) consisting of 16 patients with glioblastoma, DOC1021 appears to be safe and well tolerated, with no attributable serious adverse events observed, in addition to 93% of patients surviving 1 year. As this technology is also being evaluated in patients with pancreatic cancer (NCT04157127) and angiosarcoma (NCT05799612), it would be fascinating to understand its efficacy in treating haematological malignancies.

### 6.3 AML‐VAC‐XS15

***Mechanism**: Vaccine containing nine HLA class I‐ and class II‐restricted peptides derived from both mutated and non‐mutated AML/LPC antigens that are tumour‐exclusive resulting in CD8* ^+^ *and Th1 CD4* ^+^ *T cell responses*.

*Disease: AML*.

The poor prognosis of AML, reflected in the 5‐year survival rate of only 30%, is largely attributed to the persistence of leukaemia progenitor/stem cells (LPCs), which lead to MRD‐driven relapse [65]. Thus, elimination of such cells is imperative for long‐term survival. Despite the revolutionary success of T cell‐based immunotherapy, there remains a crucial need to reduce off‐target effects and to sustain long‐term effective anti‐tumour immune responses [66, 67]. A promising approach with minimal side effects to achieve this goal is peptide‐based immunotherapy, which seeks to provoke a T cell response against tumour antigens (TAs) displayed on the surface of tumour cells through human leukocyte antigen (HLA) molecules, facilitated by therapeutic vaccination [68, 69]. These TAs may originate from tumour‐specific mutations or from non‐mutated gene and protein products that are differentially expressed and presented in tumour cells [70]. However, previous peptide vaccination trials have struggled to produce broad clinical responses due to issues related to antigen selection, adjuvant formulations, and treatment timing [71, 72, 73].

To address such challenges, the AML‐VAC‐XS15 vaccine has been developed. The vaccine contains nine HLA class I‐ and class II‐restricted peptides derived from both mutated and non‐mutated AML/LPC antigens. The peptides underwent stringent selection through immunopeptidomic profiling of primary AML samples, which were compared with a benign immunopeptidome database, ensuring that the selected peptides are naturally presented on AML cells and are tumour‐exclusive. Importantly, the selected neoepitopes include two HLA class I‐restricted peptides from a common AML mutation in NPM1, and an HLA class II‐restricted peptide from a mutation in isocitrate dehydrogenase 2 (IDH2) (R140Q), both of which have been shown to induce memory T cell responses in AML patients [74]. The vaccine is formulated with a potent adjuvant system, consisting of the toll‐like receptor 1/2 (TLR1/2) agonist XS15, a water‐soluble derivative of Pam3Cys, and emulsified in Montanide ISA 51 VG. This optimal delivery system ensures sustained local immune stimulation at the injection site and robust CD8^+^ and Th1 CD4^+^ T cell responses after a single dose [75]. AML‐VAC‐XS15 is currently being evaluated in a Phase I clinical trial (NCT06252584) for MRD‐positive or MRD‐negative AML patients in complete cytological remission following first‐line therapy. Patients will receive two vaccinations, spaced 6 weeks apart. Following these doses, the immune response will be assessed via interferon‐gamma (IFN‐γ) ELISPOT, and a booster vaccination may be administered 3 months after the second dose [76]. Whether this peptide‐based strategy will be effective in eradicating MRD and preventing relapse in AML patients remains to be determined.

## 7 Antibody‐Based Therapies

The concept of “magic bullets” for targeted cancer therapy, proposed by Paul Ehrlich in the early 20th century, evolved with the development of monoclonal antibodies (mAbs) [77]. These antibodies, like trastuzumab and rituximab, target specific tumour antigens, but their limited cytotoxicity prompted the creation of bispecific antibodies (BiAbs) and bispecific T cell engagers (BiTEs) [78, 79, 80].

### 7.1 CLN‐049

*Mechanism: BiAb targeting both FLT3 on AML cells and CD3 on T cells*.

*Disease: AML, MDS*.

CLN‐049 is a highly engineered, T cell engaging BiAb targeting both fms related receptor tyrosine kinase 3 (FLT3) on AML cells and CD3 on T cells. The antibody features a symmetric human IgG1 backbone with two FLT3‐binding Fab arms and two CD3‐binding domains, which are functionally monovalent and fused to the IgG as a single‐chain structure. Its Fc domain is silenced, preventing unwanted activation of Fc receptors, ensuring targeted activity with minimal off‐target immune responses. CLN‐049 redirects T cells to FLT3‐expressing AML cells, enabling T cell mediated cytotoxicity. The potent cytotoxicity of CLN‐049 is demonstrated by its subnanomolar EC_50_ values, which are largely independent of FLT3 expression or mutational status, highlighted by the complete lysis of cells expressing less than 300 FLT3 receptors. Thus, it potentially offers a broader application across different patient populations. The specificity of CLN‐049 is illustrated by the lack of significant T cell activation or cytokine release in the absence of target cells, and normal FLT3‐expressing cells such as DCs in peripheral blood or CD34^+^ progenitor cells in bone marrow samples being unaffected [81]. These characteristics contrast with other bispecific antibodies targeting broader myeloid markers, like CD33, which can result in significant toxicity due to the widespread expression of these antigens on normal myeloid cells [82]. Additionally, CLN‐049 activity is unaffected by soluble FLT3 ligand (sFLT3L) or soluble FLT3 (sFLT3), even at supraphysiological levels. This is due to the two FLT3‐binding Fab arms, which promote higher avidity binding to the membrane‐proximal extracellular domain of FLT3 on AML cells. The safety and preliminary efficacy of CLN‐049 is currently being evaluated in a Phase I clinical trial (NCT05143996) in patients with r/rAML and high‐risk MDS.

### 7.2 Glofitamab

*Mechanism: BiAb targeting both CD20 on B cells and CD3 on T cells*.

*Disease: B‐NHL*.

**FIGURE 3:**
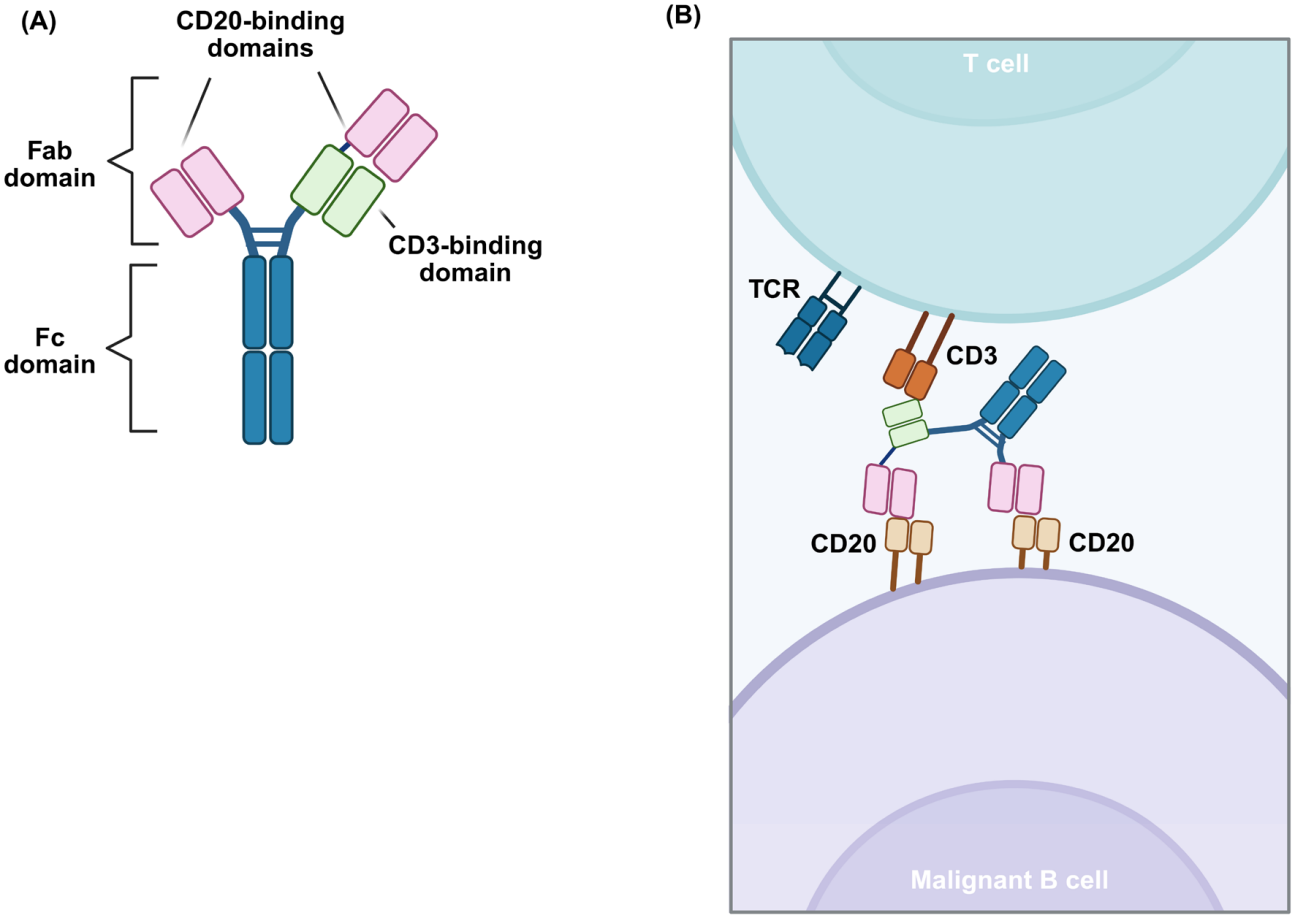
Structure and mechanism of action of glofitamab. (A) Structure of glofitamab (B) The 2:1 configuration of glofitamab enables the bivalent binding to CD20 on B cells and monovalent binding to CD3 on T cells. Fab, fragment antigen‐binding; Fc, fragment crystallizable; TCR, T cell receptor complex.

While CAR‐T therapies have shown great potential in the treatment of diffuse large B cell lymphoma (DLBCL), there are significant challenges related to manufacturing complexity, logistical constraints, and patient eligibility that restrict its broader use [83]. Glofitamab (Figure 3) is a bispecific full‐length antibody with a novel 2:1 format, where one arm selectively binds CD3 on T cells and the other arm bivalently binds to CD20 on B cells. The CD3‐binding region is linked to one of the CD20‐binding regions in a head‐to‐tail configuration through a flexible linker. This structural design enhances efficacy through optimal T cell activation and reduces the interference from competing antibodies targeting CD20, such as rituximab, offering the possibility of pre‐treatment or combination treatment with these agents. The 2:1 design promotes the engagement of both immune effector cells and tumour cells, leading to more effective elimination of tumour cells. Preclinical data from in vivo DLBCL models have demonstrated that glofitamab had greater efficacy and potency when compared to other CD20 x CD3 BiAbs, positioning it as a potential leader in this therapeutic field. Furthermore, glofitamab requires being less frequently administered when compared to other bispecific T cell engagers that lack an Fc domain due to having a half‐life of approximately 10 days [84].

A Phase I study (NCT03075696) treating heavily pretreated B cell non‐Hodgkin lymphoma (B‐NHL) patients with glofitamab has demonstrated an ORR of 49%, with 39% achieving CR, including 81% of patients who relapsed after CAR‐T therapy. A UK retrospective analysis of 59 patients further confirmed these findings, with a 22% complete metabolic response (CMR) and durable responses, even in prior CAR‐T‐treated patients. Glofitamab exhibited a tolerable safety profile, with low‐grade CRS and 31% severe neutropenia. In the Phase III trial (NCT04408638), glofitamab plus gemcitabine‐oxaliplatin (GemOx) showed a clear OS benefit when compared to rituximab plus GemOx, with a median OS of 25.5 months versus 12.9 months. This combination demonstrated a manageable safety profile, with 44% of patients experiencing CRS, mostly low‐grade, highlighting glofitamab's treatment potential in transplant‐ineligible patients [85].

### 7.3 Epcoritamab

*Mechanism: BiAb targeting both CD20 on B cells and CD3 on T cells*.

*Disease: CLL, SLL, RS*.

Richter's syndrome (RS) is a highly aggressive transformation of CLL, most often into DLBCL, and occurs in 2%–15% of CLL patients. The poor prognosis, reflected by a 20% CR rate and long‐term survival below 20% post‐chemoimmunotherapy, has driven the development of several novel therapeutic agents [86]. One such agent, epcoritamab, is a subcutaneously administered, IgG1‐based bispecific antibody that simultaneously binds CD3 on T cells and CD20 on B cells. This dual‐targeting mechanism facilitates T cell activation and proliferation, leading to direct cytotoxicity against CD20^+^ tumour cells. Subcutaneous administration offers the advantage of extending the drug's half‐life while minimising excessive cytokine release, thereby enhancing overall tolerability. Unlike conventional monoclonal antibodies, epcoritamab's modified Fc region prevents Fcγ receptor‐mediated interactions, reducing off‐target T cell activation. In patients with r/rDLBCL, epcoritamab demonstrated rapid and sustained reduction in peripheral B cells, activation of CD4^+^ and CD8^+^ T cells, in addition to increases in IFN‐γ, interleukin 6 (IL‐6) and tumour necrosis factor‐alpha (TNF‐α) [87].

Preliminary results from the Phase Ib/II clinical trial (NCT04623541) evaluating epcoritamab for the treatment of patients with RS have demonstrated high ORRs and CRRs of 60% and 50%, respectively. Interestingly, most patients presented with tumour reduction at the first assessment, which occurred at 6 weeks, with the most common TRAEs being CRS, occurring in 90% of patients, although all cases of CRS were resolved without discontinuation of epcoritamab.

### 7.4 ICT01

***Mechanism**: mAb targeting* *BTN3A*.

*Disease: bladder, breast, colon, gastric, melanoma, ovarian, prostate, PDAC, AML, FL, HNSCC*.

Gamma delta T (γδ T) cells are a unique subset of T cells that exhibit characteristics of both the innate and adaptive immune systems [88]. In peripheral blood, γδ T cells account for 1%–5% of the total CD3^+^ T cells in humans. These cells are activated in an MHC‐independent manner and are particularly effective against cancerous or pathogen‐infected cells, as they can identify tumour‐specific molecular patterns such as an accumulation of phosphoantigens (pAgs) [89]. When activated, γδ T cells release pro‐inflammatory cytokines, such as IFN‐γ and TNF‐α and exhibit cytotoxic activity through mechanisms involving perforin and granzymes [90]. In cancer, γδ T cells are often represented by tumour‐infiltrating lymphocytes (TILs) and have therefore been associated with a favourable prognosis in several malignancies, including leukaemia, breast cancer, and melanoma [90, 91]. Despite their cytotoxic potential, harnessing these cells for cancer treatment has not yet come to fruition [92].

**FIGURE 4:**
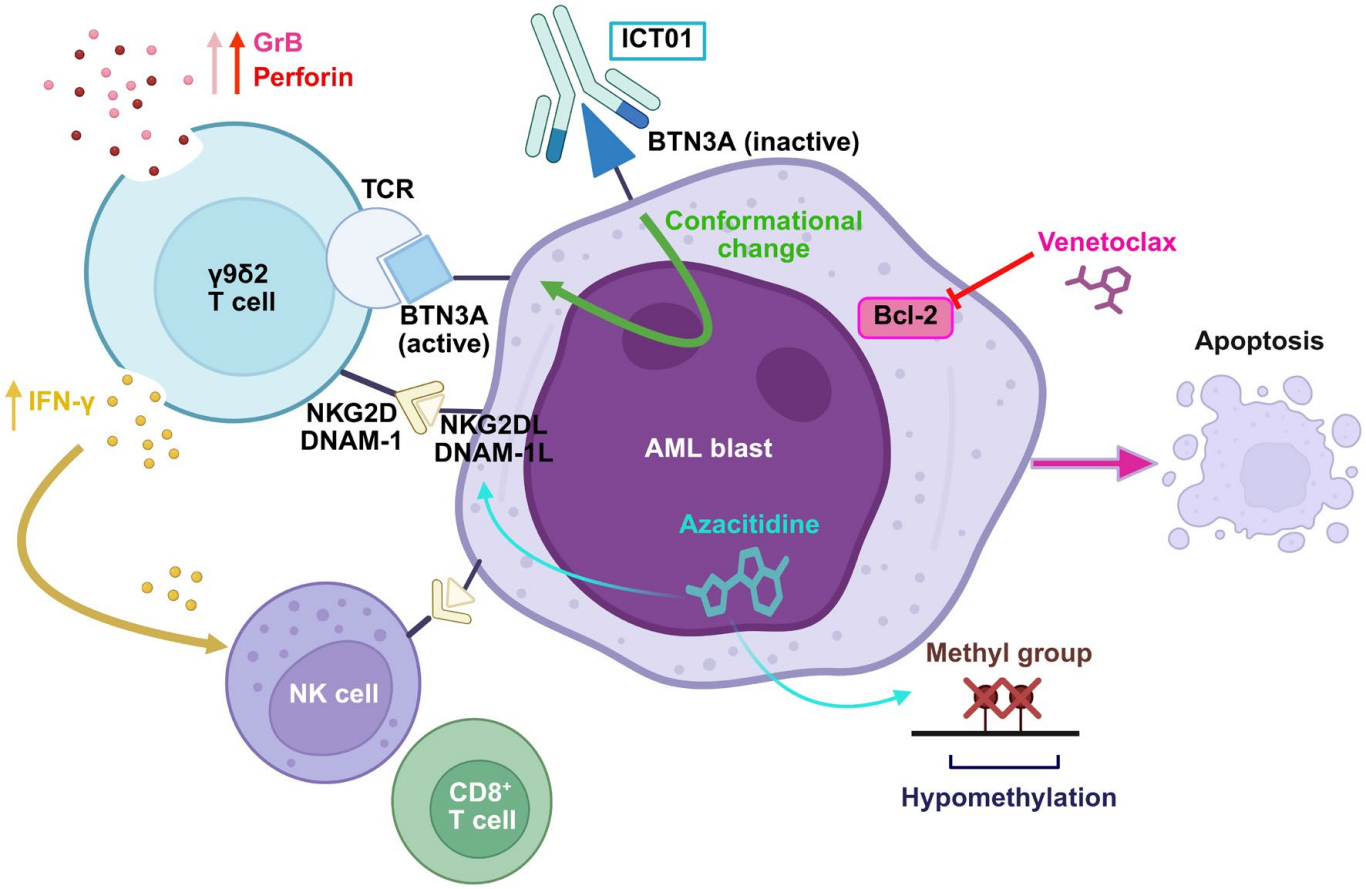
ICT01‐induced activation of γ9δ2 T, NK and CD8^+^ cells through BTN3A synergises with azacitidine‐venetoclax treatment to kill AML cells. Following the binding of ICT01 to BTN3A on the surface of AML blasts, a conformational change occurs resulting in BTN3A being in an ‘active’ state. The now active BTN3A directly activates γ9δ2 T cells through its engagement with the TCR, resulting in the killing of AML blasts via the release of GrB and perforin. The subsequent activation of NK and CD8^+^ T cells by the activation of receptors such as NKG2D and DNAM‐1, and the release of IFN‐γ enhances the immune‐mediated targeting of AML cells. The inhibition of Bcl‐2 by venetoclax overcomes the previously recognised resistance of AML blasts to GrB/perforin, thus enhancing the anti‐leukaemic efficacy of activated γ9δ2 T cells. In addition to promoting hypomethylation and the formation of double‐strand DNA breaks, azacitidine heightened immune‐effector‐cell recognition of AML blasts through induction of secondary stress ligands such as NKG2DL. BTN3A, butyrophilin 3A; TCR, T cell receptor complex; NKG2D, natural killer group 2, member D; DNAM‐1, DNAX accessory molecule‐1; NKG2DL, NKG2D ligand; DNAM‐1, DNAM‐1 ligand; GrB, granzyme B; IFN‐γ, interferon‐gamma; Bcl‐2, B cell lymphoma‐2, AML, acute myeloid leukaemia.

ICT01 (Figure 4) is a first‐in‐class humanised monoclonal antibody designed to target butyrophilin 3A (BTN3A), a type I transmembrane protein located on the surface of AML blasts and is involved in the activation of γ9δ2 T cells. Following the binding of ICT01 to BTN3A, a conformational change occurs resulting in BTN3A being converted to an ‘active’ state. This activity triggers BTN3A to interact with the T cell receptor of γ9δ2 T cells, resulting in an immune cascade involving the production of granzymes and perforins, ultimately causing cell death of AML blasts. Additional immunomodulatory effects include activation of granulocytes and NK cells and elevated release of IFN‐γ. These overall immune effects initiated by ICT01 amplify γ9δ2 T cell function through activating co‐stimulatory immune processes through receptors such as natural killer group 2, member D (NKG2D), DNAX accessory molecule‐1 (DNAM‐1), and intercellular adhesion molecule 1 (ICAM1), further enhancing immune‐mediated blast killing [92].

Preclinical findings have demonstrated the synergistic potential of ICT01 when combined with venetoclax and azacitidine in significantly improving therapeutic outcomes. Through Bcl‐2 inhibition, venetoclax alleviated the resistance of AML cells to granzyme B/perforin‐mediated killing, amplifying the anti‐leukaemic effects of ICT01‐activated γ9δ2 T cells, NK cells, and CD8 ^+^ T cells. Not only is azacitidine directly involved in killing AML cells through DNA hypomethylation, but it also improves immune‐effector cell recognition of AML cells. For instance, azacitidine induced the expression of secondary stress ligands such as NKG2D ligand (NKG2DL) on AML cells. Overall, it improves the recognition and targeting of AML blasts by immune cells, enhancing the efficacy of both ICT01 and venetoclax. These findings strongly supported investigating the combination of ICT01 with venetoclax and azacitidine in patients with AML. ICT01 is currently being evaluated in a Phase I clinical trial (NCT04243499) in combination with venetoclax and azacitidine to treat adults with newly diagnosed AML. The combination therapy so far has shown to be safe and effective, indicated by a CR of 75% and a CR/CRi of 94%, even in patients with an adverse‐risk ( *TP*53^MT^ , ASXL1^MT^ ) and intermediate‐risk (NPM1^MT^ , IDH1/2^MT^ ) genetic profile, suggesting that the combination treatment is efficacious across several subtypes of AML. Importantly, ICT01 rapidly activated γ9δ2 T cells and NK cells, which was sustained in the high dose arm [92, 93].

### 7.5 INCA033989

*Mechanism: mAb targeting mutCALR*.

*Disease: MPN*.

Despite the acquired somatic unique *JAK2* V617F mutation being responsible for the development of MPN in 60% of patients with essential thrombocythemia (ET), and 55% of patients with MF, mutations (+1 frameshifts) in calreticulin (mutCALR) are the second most prevalent and occur in 25% and 35% of patients diagnosed with ET and MF, respectively [94, 95, 96]. Both type I (CALRdel52) and type II (CALRins5) mutations in CALR generate a neoepitope on the C‐terminus of the protein, which abnormally interacts with the thrombopoietin receptor (TPOR/MPL), activating it in the absence of TPO ligand [97, 98, 99]. Importantly, the pathogenic effects of mutant CALRs are requiring transport of the complexes with TPOR at the cell‐surface, thus, making the antigenic new tail of the mutant CALR exposed at the cell‐surface [100]. Overall, this results in constitutive activation of the Janus kinase 2 (JAK2)—signal transducer and activator of transcription (STAT) signalling pathway, driving pathological proliferation of HSCs, in particular megakaryocytes [101]. Current treatments for patients with mutated CALR include JAK2 inhibitors or cytoreductive therapies. While both primarily provide symptomatic relief, neither offers selectivity for the mutated clone, limiting the potential for CRs [102]. The development of novel compounds that specifically target cells expressing mutCALR, would greatly enhance the elimination of neoplastic cells while sparing normal haematopoiesis.

INCA033989 is a human IgG1 monoclonal antibody that selectively binds to the unique C‐terminus of the mutCALR protein, blocking the activation of TPO‐R (MPL) ultimately inhibiting the disease‐driving JAK–STAT signalling cascade and effectively blocking cytokine‐independent proliferation. This inhibition is related to a dynamin‐dependent endocytosis of the INCA033989/mutCALR/TPOR complex, reducing the presence of active receptors on the cell surface. Notably, INCA033989 functions independently of Fc‐mediated immune functions such as antibody‐dependent cellular cytotoxicity (ADCC) or complement‐dependent cytotoxicity (CDC), as the Fc domain has been designed to silence these functions, thus reducing the risk of immune‐related side effects. The primary appeal of INCA033989, lies in its ability to selectively bind to HSPC clones expressing mutCALR, sparing normal, wild‐type CALR‐expressing cells, thus, distinguishing itself from previously approved treatments for MPNs. Preclinical models have demonstrated potent and selective activity of INCA033989, resulting in the reduction of mutCALR‐positive platelets and megakaryocytes, in addition to the leukaemic initiating cells without impacting JAK2 V617F mutated cells, whilst preserving bone marrow cellularity and normal haematopoiesis [103]. Findings from mouse models have shown promising pharmacokinetic properties, with INCA033989 demonstrating a half‐life of 128 h and high plasma concentration exceeding the 90% inhibitory concentration required for effective in vivo activity. Overall, these data suggest a therapeutic window that is suitable for patient treatment.

Potential limitations would be that INCA033989 demonstrates preferential binding to the CALRdel52 mutation, suggesting that higher doses may be required for patients with CALRins5 mutations to achieve an effective response. Additionally, the pro‐inflammatory bone marrow microenvironment that is observed in MF, including the dense number of megakaryocytes and fibrosis, may hinder antibody access to disease‐initiating HSCs. The promising preclinical studies and novel antibody design have advanced INCA033989 into Phase I clinical trials (NCT06034002, NCT05936359) for patients with MF and ET [103, 104].

### 7.6 JNJ‐88549968

*Mechanism: BiAb targeting mutCALR on MPN cells and CD3 on T cells*.

*Disease: MPN*.

The bispecific antibody JNJ‐88549968 (CALRmutxCD3) from Johnson & Johnson targeting the mutated CALR and aiming to eradicate the MPN clone is now in clinical trials (NCT06150157). The antibody targets the C‐terminus novel sequence of all mutant CALR variants in MPN and CD3 on T cells, thus engaging CD8 T cells into killing cells that expose on the cell‐surface the mutated CALR tumour antigen. Preclinical work indicated specificity for mutated cells in whole blood assays and cell line models [105, 106].

Finally, targeted efforts are underway to harness CAR‐T cells that recognise the mutated CALR sequence [107].

### 7.7 MYTX‐011

*Mechanism: ADC targeting c‐MET*.

*Disease: NSCLC*.

The mesenchymal‐epithelial transition factor (c‐MET) also known as hepatocyte growth factor receptor (HGFR), is a receptor tyrosine kinase (RTK) that plays essential roles in embryogenesis, organ development, and tissue regeneration. Following the binding of its ligand, hepatocyte growth factor (HGF), c‐MET becomes activated resulting in receptor dimerization and autophosphorylation. Ultimately, activating a cascade of signalling pathways associated with migration, proliferation, survival, and angiogenesis [108]. Indeed, dysregulation of c‐MET occurs during tumorigenesis which can be caused by overexpression of HGF, mutations in c‐MET, and MET gene amplification, all of which result in sustained activation of c‐MET signalling. In solid tumours, in particular, in non‐small cell lung cancer (NSCLC), gastric cancer, and renal carcinoma, c‐MET is frequently mutated or overexpressed, which is associated with poor prognosis and resistance to standard‐of‐care therapies [109]. As a result, c‐MET has become a promising therapeutic target, with several antibody‐drug conjugates (ADCs) in early clinical development [110, 111].

MYTX‐011 represents a novel advancement in ADC technology, leveraging the engineering of the antibody component to enhance tumour targeting and therapeutic efficacy in cancers expressing c‐MET. The novelty of MYTX‐011, stems from the incorporation of pH‐dependent binding into an anti‐c‐MET antibody, which allows for selective release of its cytotoxic payload, valine citrulline monomethyl auristatin E (vcMMAE), within the acidic microenvironment of tumour cells [112]. This design overcomes the common limitation of conventional ADCs, which often show reduced efficacy in tumours with low or heterogeneous target expression [113]. In a physiological environment of a pH of 7.4, the high‐affinity binding of MYTX‐011 to c‐MET is maintained, whilst in acidic conditions such as in endosomes (pH of approximately 5.4), MYTX‐011 rapidly dissociates from c‐MET. This dissociation significantly enhances the internalisation of MYTX‐011 into c‐MET^+^ tumour cells. The pH‐dependent nature of the interaction mitigates recycling of the receptor, contributing to greater accumulation of the ADC within the cells and consequently increased cytotoxicity [112].

Preclinical work demonstrated that MYTX‐011 had substantially improved internalisation and potency compared with a non–pH‐engineered parent ADC, particularly in cells expressing moderate to low levels of c‐MET. In vivo, MYTX‐011 exhibited enhanced antitumor efficacy across several NSCLC xenograft models, including those with epidermal growth factor receptor (EGFR) and kirsten rat sarcoma virus (KRAS) mutations, and those that expressed c‐MET at various levels. Notably, MYTX‐011 demonstrated improved therapeutic activity compared to the parent ADC and a benchmark anti‐c‐MET ADC. The pharmacokinetic profile of MYTX‐011 was evaluated in cynomolgus monkeys and demonstrated a significantly extended half‐life of 158 h, indicative of reduced target‐mediated drug disposition (TMDD), ultimately broadening the therapeutic index of MYTX‐011 [107]. These encouraging preclinical findings have led to the initiation of a Phase I clinical trial (NCT05652868) treating locally advanced or metastatic NSCLC with MYTX‐011. The most recent results from the trial indicate that MYTX‐011 is stable, with minimal separation of ADC and total mAb concentrations. Regarding safety, 32% of patients experienced Grade ≥ 3 TRAEs, although the incidence of peripheral neuropathy and hematologic toxicities were low. MYTX‐011 was well tolerated, with no dose‐limiting toxicities reported during dose escalation [114].

Interestingly, it has been suggested that the survival of CD5^+^ B cells from patients with B‐CLL involves altered c‐MET expression, with higher expression of c‐MET compared to normal B cells. Indeed, B‐CLL patients also have increased HGF in their serum. Together, HGF/c‐MET signalling was shown to activate pro‐survival mediators including increasing the expression of B cell lymphoma‐extra large (Bcl‐XL) and phospho‐Akt, whilst negatively regulating the pro‐apoptotic Bcl‐2 associated agonist of cell death (Bad) protein [115, 116, 117]. Since c‐MET appears to be involved in the survival of B‐CLL cells, it would be interesting to discover whether these patients would benefit from MYTX‐011, especially those who have limited treatment options.

Despite the emerging interest in developing novel ADCs to target leukaemia, such as the recent first‐in‐class anti‐CD33 x anti‐CD7 bispecific ADC, BVX001, gemtuzumab ozogamicin (GO) and inotuzumab ozogamicin (IO) currently remain the only ADCs to be granted FDA approval [118]. Despite their approval, the success of these therapies has been limited by significant toxicity concerns such as prolonged cytopenias and liver damage, including sinusoidal obstruction syndrome (SOS) or veno‐occlusive disease (VOD) [119]. By adopting a similar approach to MYTX‐011, such as screening for anti‐c‐MET antibody mutants that selectively lose binding under acidic conditions and are rapidly internalised, could help broaden the therapeutic window of ADCs in leukaemia.

## 8 Concluding Remarks

During 2024–2025, significant advancements have been made in the development of novel technologies/approaches within the biotechnology and pharmaceutical industries, particularly with respect to the treatment of patients with haematological malignancies. A primary focus has been overcoming previous resistance mechanisms through innovative drug design, while simultaneously minimising high‐grade toxicities. This has resulted in the emergence of highly targeted therapeutic strategies with the potential for long‐term efficacy. As the treatment landscape for haematological cancers continues to evolve, it will be critical to stratify patient demographics to identify those most likely to benefit from specific treatments. For approaches that prove successful, refinement and further development will follow, and additional research into those mechanisms will be necessary. The need to integrate multimodal approaches, such as combining immunotherapy with small molecules, will be pivotal for achieving more effective and durable cancer therapies.

Ongoing advances in our understanding of the complexities of gene regulation, including the role of IGDs, have facilitated the exciting finding that genes within these domains can be modulated in a tuneable manner by harnessing the potential of ECs. This development raises the intriguing possibility of applying this technology, in conjunction with other advancements, such as harnessing the versatile ADC design used in MYTX‐011, to treat patients with an unfavourable prognostic background or those exhibiting low or fluctuating levels of tumour specific antigens.

## Author Contributions

**Akbar M. Shahid:** conceptualization (equal), data curation (lead), formal analysis (lead), investigation (lead), visualization (lead), writing – original draft (lead), writing – review and editing (equal). **William Vainchenker:** conceptualization (supporting), writing – review and editing (supporting). **Stefan N. Constantinescu:** conceptualization (equal), funding acquisition (lead), resources (lead), supervision (lead), writing – review and editing (equal).

## Conflicts of Interest

S.N.C. is a founder of MyeloPro GmbH, Vienna, Austria. W.V. has received support for research from Incyte Corporation.

## Data Availability

Data sharing not applicable–no new data generated, or the article describes entirely theoretical research.
